# Subgingival Microbiome Profiles in Bulgarian Children Aged 10–14 with Gingival Inflammation and Healthy Periodontium: A Targeted PCR Study

**DOI:** 10.3390/microorganisms13071656

**Published:** 2025-07-14

**Authors:** Hristina Tankova, Nadezhda Mitova

**Affiliations:** Department of Pediatric Dental Medicine, Faculty of Dental Medicine, Medical University, 1431 Sofia, Bulgaria; n.mitova@fdm.mu-sofia.bg

**Keywords:** microbiome, children, periodontopathogens, gingival inflammation

## Abstract

The subgingival microbiome is a critical component of the oral microbiota and plays a central role in pediatric periodontology. This study investigated differences in periodontopathogen profiles in children with gingival inflammation compared to healthy controls using real-time PCR, with a focus on the microbial complexes defined by Socransky. A total of 73 children (ages 10–14) underwent comprehensive periodontal assessment, including assessments of general health status, the O’Leary hygiene index (HI), gingival condition, and the papillary bleeding index (PBI). Subgingival plaque samples were analyzed using real-time PCR to identify key bacterial species associated with gingival health and disease. Highly pathogenic periodontopathogens such as *Aggregatibacter actinomycetemcomitans*, *Porphyromonas gingivalis*, *Treponema denticola*, and *Eubacterium nodatum* were absent in healthy subjects. In contrast, *Tannerella forsythia* was significantly more frequently detected in children with gingival inflammation (*p* < 0.05). The most abundant species in the inflammation group were *Prevotella intermedia* and *Capnocytophaga gingivalis.* Children with gingival inflammation exhibit a distinct subgingival microbiome profile characterized by an increased presence of specific periodontopathogens, including a higher prevalence of red complex species as defined by Socransky. However, the cross-sectional nature of this study limits the ability to establish causal relationships.

## 1. Introduction

The human microbiome represents an ecosystem of microorganisms that inhabit the human body and interact with each other in symbiosis [1]. The formation of the modern microbiome reflects millions of years of co-evolution between humans and microorganisms. Over time, both have mutually adapted, and the microbiome now plays a crucial role in both our health and the development of pathological conditions [2].

In 2018, the American Academy of Periodontology (AAP) and the European Federation of Periodontology (EFP) adopted the latest classification of periodontal diseases and peri-implantitis conditions [3]. The essence of the new concept, incorporated into the modern classification of periodontal diseases, focuses on local risk factors (some of which are microbial factors) and the inflammation they cause in the gingiva. Experts agreed that the presence of “limited gingival inflammation” (with bleeding on probing (BOP)—up to 10%) is still not sufficient to classify a patient as a “gingivitis case”, but this condition is of interest from a preventive point of view and is reflected in the latest classification as “site-specific” inflammation (gingivitis site) [3,4]. From the perspective of pediatric dentistry and the numerous data in the global literature on the widespread occurrence of gingival inflammation in adolescents, the relationships between microorganisms specific to this transition process between health and inflammation are of particular interest [5,6,7]. This is especially true between the ages of 10 and 14, when there is an active transition from primary to permanent dentition, poor oral hygiene habits, and hormonal changes and when periodontal space needs time to stabilize [8,9].

The leading etiological factor for periodontal diseases is dental biofilm and its interaction with the adolescent immune system. It has been experimentally established that there are specific interactions between microorganisms in the dental biofilm and the gingival tissues of the host [10].

Through microscopic studies, researchers have found that the structure of the dental biofilm follows a strictly defined order, in which certain microorganisms dominate or are in close association with other microorganisms, united in microbial associations [11,12]. Driven by these results, Socransky conducted his own study on adult patients, isolating 13,261 subgingival samples. The results revealed five closely related groups of microorganisms based on their simultaneous presence in a specific niche. The yellow, green, and purple complexes, or the so-called initial colonizers, precede the colonization of Gram-negative microorganisms from the red and orange complexes [13]. Species from the orange and red complexes are primarily associated with the initiation and progression of periodontal diseases. As early as 1996, *Aggregatibacter actinomycetemcomitans*, *Porphyromonas gingivalis*, and *Treponema denticola* were identified by the AAP as the so-called “major periodontal pathogens” [14].

While next-generation sequencing (NGS) methods offer the comprehensive profiling of the oral microbiome, this study employs a targeted multiplex real-time PCR panel focusing on nine clinically relevant periodontopathogens. This approach balances clinical applicability, reproducibility, and cost-efficiency, enabling focused investigation within a pediatric population where standardized diagnostics are crucial. Although NGS provides broader microbial insights, the selected panel represents well-established key pathogens associated with periodontal disease progression, facilitating reliable quantification and interpretation in this specific clinical context [15,16].

A review of the global literature shows that subgingival microorganisms from the red complex, as well as *A. actinomycetemcomitans*, are also isolated in systemically healthy children with a healthy periodontium, although they do not induce inflammation in the gingival tissues of these children, according to Socransky. Predictably, in children with inflammatory destructive changes in the periodontium, subgingival microorganisms are present in greater numbers and in more complex interactions [17].

Recent studies employing next-generation sequencing have expanded the understanding of pediatric oral microbial diversity and its association with periodontal health and disease [18,19]. These findings emphasize the complex microbial dynamics in children but also highlight the need for targeted, clinically applicable diagnostic tools. Our study complements these data by focusing on a clinically validated PCR panel to explore microbial profiles in children with gingival inflammation.

Subgingival flora has also been studied in children with systemic diseases, where the results show significant diversity. In these children, the most commonly isolated periodontal pathogens are *P. gingivalis* and representatives of the green and yellow complexes, according to Socransky. The diversity is due to the fact that, depending on the child’s systemic disease, certain periodontal pathogens predominate over others, depending on the conditions provided by the oral cavity in the respective disease. Socransky asserts that in children with periodontitis and the presence of systemic diseases modifying the gingival environment, representatives of the red and yellow complexes are most commonly isolated [20].

In recent years, there has even been a revision of the Socransky complexes, with authors grouping specific subgingival microorganisms found in certain gingival statuses into bacterial clusters. In health, gingivitis, peri-implant mucositis, periodontitis, peri-implantitis, necrotizing periodontitis, and molar–incisor periodontitis. These authors studied adult patients and found that, in the health cluster, *P. gingivalis*, *Prevotella intermedia*, and *Tannerella forsythia* were included, although they were isolated in relatively low relative proportions. In the gingivitis cluster, the same microorganisms were isolated but at a higher frequency, and along with them, *Fusobacterium* spp., *Treponema* spp., and *A. actinomycetemcomitans* were isolated. In the periodontitis cluster, the representatives of the red complex were found together with *A. actinomycetemcomitans*, *P. intermedia*, and *Fusobacterium* spp. [21].

It is noticeable that studies in the literature are focused on examining the subgingival microbiome in periodontally healthy children and those with inflammatory destructive changes in the periodontium. In-depth studies on children with gingivitis are relatively rare. This provides a strong rationale for investigating the current state of the subgingival microbiome in children in the context of their periodontal status.

The novelty of this study lies in its focus on children with gingival inflammation, a transitional periodontal condition that is less studied compared to both healthy children and those with established periodontitis. Investigating the subgingival microbiota in this specific cohort provides important insights into early microbial changes associated with gingival inflammation and may contribute to improved early diagnosis and preventive oral health strategies in pediatric populations [15,16].

The aim of this study was to investigate and compare key microorganisms of the subgingival microbiota in children with healthy periodontium and children with gingival inflammation.

## 2. Materials and Methods

### 2.1. Study Design

This cross-sectional observational targeted study was conducted between January 2021 and May 2021 within the Department of Pediatric Dental Medicine, Medical University, Sofia, Bulgaria. It was conducted according to the guidelines of the Declaration of Helsinki and was approved by the Ethics Committee of the Medical University, Sofia (KENIMUS), protocol № 12/14 May 2020.

The inclusion and exclusion criteria for participation were, respectively, as follows: systemic health and the absence or presence of gingival inflammation; the presence of a systemic disease or use of medications that may affect gingival status, the presence of fixed orthodontic appliances, and the use of antibiotics in the last three months [22].

Before conducting this study, a power analysis was performed to determine the appropriate sample size. We selected a power of 0.80, with a significance level of 0.05 and an expected medium effect size (Cohen’s d = 0.5). The power analysis indicated that a sample size of 50 children would be sufficient to detect statistically significant effects, if they exist. This approach ensured that this study was adequately powered to identify real differences while minimizing the likelihood of Type II errors.

A total of 80 children were examined. Among them, 8 were excluded because of antibiotic therapy or fixed orthodontic appliances. At the end of the recruitment period, 73 systemically healthy children aged 10 to 14 years met the inclusion criteria and participated in this study.

The study participants were children in early puberty, a developmental stage associated with significant changes in oral microbiota composition. Informed consent was obtained from the legal guardians of all subjects involved in this study.

Gingival inflammation was assessed in accordance with the 2017 World Workshop Classification of Periodontal Diseases, specifically incorporating the concept of site-specific inflammation. Although this classification defines bleeding on probing (BoP) as the standard diagnostic criterion, we employed the papillary bleeding index (PBI) [23] as a validated and age-appropriate clinical index to quantify the extent and severity of inflammation in children. While not a substitute for BoP, PBI allowed consistent and objective assessment of gingival status in this pediatric cohort. Oral hygiene was evaluated using the O’Leary Plaque Index (HI) [24].

Gingival inflammation was diagnosed based on the following criteria:

- The presence of provoked gingival bleeding in more than 10% of sites (PBI > 10%).
- Less than 50% of plaque-free surfaces (HI < 50%).

After selection, the children were divided into two groups:

- Children with healthy periodontium (PBI prevalence up to 10%).
- Children with gingival inflammation (PBI prevalence over 10%).

### 2.2. Clinical Examination of Children

Each child underwent a clinical periodontal examination in four steps:

First Step: General Health Status Assessment—Anamnesis was used to gather information on the child’s overall health.

Second Step: Oral Hygiene Status Assessment—We used the O’Leary hygiene index (HI), which measures the percentage of plaque-free surfaces. All fully erupted permanent teeth were stained with a plaque-disclosing solution (2Tone Disclosing Solution from Young Dental). Each tooth was divided into four surfaces (vestibular, distovestibular, mesiovestibular, and oral), with plaque presence recorded as “+” and plaque absence as “−”.

Third Step: Assessment of Teeth and Gingiva—This included the registration of dental status, the condition of soft tissues, and the presence of orthodontic anomalies.

Forth Step: Gingival Sulcus Assessment—Evaluated by measuring gingival sulcus depth and the papillary bleeding index (PBI) with a WHO 621 manual periodontal probe [25]. For the PBI, four papillae were examined, two vestibular and two oral, on all permanent teeth. The probe is inserted into the gingival sulcus and is withdrawn in a distal direction towards the apex of the distal papilla and subsequently in a medial direction towards the medial papilla. The same is performed from the oral side. The index is calculated based on the presence of bleeding at the probing sites and represents the relative proportion of bleeding papillae to all examined papillae.

### 2.3. Real-Time PCR Method for Identifying Subgingival Microorganisms

To identify subgingival microorganisms and determine their quantities, the real-time PCR method was used.

For the purposes of this study, nine control strains of subgingival microorganisms were analyzed in a pooled sample: *Aggregatibacter actinomycetemcomitans*, *Porphyromonas gingivalis*, *Treponema denticola*, *Tannerella forsythia*, *Prevotela intermedia*, *Parvimonas (micromonas)micros*, *Fusobacterium nuleatum*, *Eubacterium nodatum*, and *Capnocytophaga gingivalis*.

The panel of microorganisms reflects the standard configuration of the validated diagnostic PET kit (MIP Pharma GmbH), which includes key periodontopathogenic species widely recognized in the literature, particularly from the red and orange complexes. While additional organisms may be relevant in pediatric oral microbiota, the panel was selected to ensure methodological consistency and clinical relevance across all samples.

#### 2.3.1. Methodology for Sample Collection for PCR Analysis

After evaluating the gingival status of the children, five teeth were selected (at least one fully erupted tooth from each quadrant) with the greatest probing depth and higher values of the PBI, from which a sample was taken for PCR analysis. The deeper the gingival sulcus, the better the conditions for the survival of subgingival microorganisms, which is why we chose teeth with the greatest depth and the presence of inflammation for sampling.

**Figure 1:**
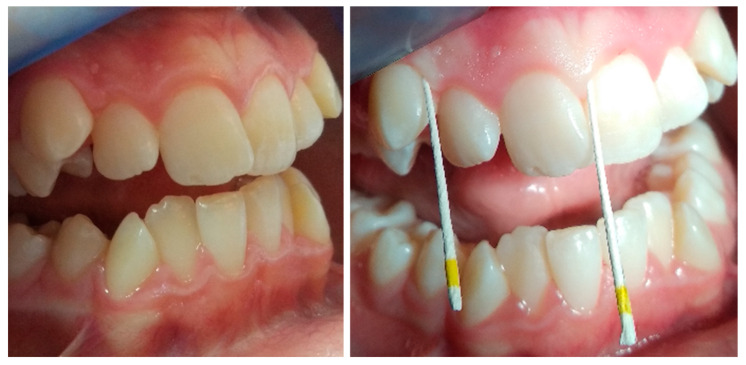
Before and during the collection of a subgingival biofilm sample for PCR analysis.

The PCR samples were taken in the morning—at least ½ hour after tooth brushing and 1 h after eating. Immediately before collection, the children rinsed their mouths with physiological saline solution, and supragingival deposits were removed using a dry sterile cotton swab. The field was isolated with a lignin roll, dried, and a sterile paper point (PET kits made by MIP Pharma GmbH) was inserted into the gingival sulcus of each selected tooth. After waiting for approximately 20 s, the paper points were collected in a transport Eppendorf tube and shipped to the laboratory in Germany (Figure 1).

#### 2.3.2. DNA Isolation and Real-Time PCR Analysis

DNA isolation was performed using the MagNA Pure 96 system (Roche Diagnostics), an automated platform designed for high-throughput nucleic acid extraction. Bacterial lysis was initiated by adding a specific lysis buffer to each sample prior to loading. The system processes up to 96 samples per run with minimal manual handling, significantly reducing the risk of contamination. Reagents (Roche Diagnostics DNA Isolation Kit for Gram-Positive and Gram-Negative bacteria) and plastic consumables were supplied in pre-packaged, barcoded cartridges. The closed-system format and full traceability ensured process reliability and reproducibility.

PCR set-up was conducted using the Hamilton Microlab STARlet IVD platform, fully validated for in vitro diagnostics. The instrument automatically pipetted and mixed the reagents and DNA samples for real-time PCR. A multiplex real-time PCR assay was developed, allowing for the simultaneous detection of up to three bacterial targets per reaction. For standard analysis, two reaction mixes were used; for extended testing (plus protocol), four mixes were applied. The PCR mix (Roche) was supplemented with manually added validated primers and probes. Barcoding was used to ensure the traceability of both reagents and plasticware.

Quantitative real-time PCR was carried out using the LightCycler 480 system (Roche Diagnostics, Basel, Switzerland). The samples were loaded into 96- or 384-well microtiter plates, depending on batch size. Reactions were performed in accordance with the manufacturer’s protocols for SYBR Green or probe-based detection. Internal standards with known DNA concentrations were included in each run for quantification and normalization purposes. Data analysis was based on amplification curve profiles and Ct values.

#### 2.3.3. Validation and Technical Details of Real-Time PCR

The Real-Time PCR analysis was conducted at an accredited diagnostic laboratory using fully automated IVD-certified systems (MagNA Pure 96 (Roche Diagnostics GmbH, Mannheim, Germany) and Hamilton STARlet IVD (Reno, NV, USA)). DNA extraction and PCR set-up were performed under highly standardized conditions using barcoded, closed-system cartridges and reaction mixes (Roche Diagnostics), minimizing contamination risk and ensuring traceability. A multiplex PCR format was used, enabling the simultaneous detection of up to three bacterial species per reaction. Although primer and probe sequences are proprietary and cannot be disclosed, their design ensures high specificity to target DNA regions of the included periodontal pathogens, and their performance has been validated internally by the manufacturer. Assay efficiency was consistently within 90–110%, and the lower limit of detection (LOD) was approximately 10^3^ genome copies/mL. Internal standards and positive controls were used in each run for calibration and quantification. Inter-assay variation remained below 5%, confirming high reproducibility. Quantitative analysis was based on fluorescence curves using SYBR Green and/or TaqMan probes, following the standard protocols of the LightCycler 480 system. Additional details are available upon request.

### 2.4. Data Analysis

The recorded data from this study were coded and entered into a computerized database, then processed using specialized IBM SPSS software, version 19.0, and MS Excel 2019. To correct for multiple comparisons, we applied the Bonferroni correction, adjusting the significance level to 0.01 based on the number of tests performed.

The Kolmogorov–Smirnov test was used to check the frequency distribution, which showed a lack of normal distribution.

Non-parametric tests were used to analyze the results.

- Descriptive Analysis: This was presented in tabular form, showing the frequency distribution of the studied characteristics based on specific indicators.
- Method for Comparing Independent Groups: Two independent sample tests were performed.
- Pearson Chi-Square Test (χ^2^): This was used to test the hypotheses of dependence between categorical variables.

## 3. Results

### 3.1. Distribution by Dentition, Gender, and Periodontal Status

The following table presents the distribution of children participating in this study by dentition, gender, and periodontal status (Table 1).

The table shows that a total of 21 out of all the examined children had mixed dentition, while twice as many had permanent dentition. In total, 40 were periodontally healthy, while 33 had gingival inflammation. There was an even distribution between the sexes in periodontally healthy and gingivitis cases, with only a statistically significant lower number of girls with gingivitis (χ^2^ = 45,685 *p* < 0.05).

### 3.2. Oral Hygiene Status (HI—Plaque-Free Surfaces) and Gingival Status—PBI (Prevalence)

The following table presents the oral hygiene status (*HI*) of the children from both study groups and the gingival status, recorded through the prevalence of provoked gingival bleeding among the examined children across different age groups (Table 2).

In children with healthy periodontium, the percentage of plaque-free surfaces is around or above 80%, indicating that plaque accumulation affects less than ¼ of the examined surfaces. In contrast, children with gingival inflammation show only 1/3 of surfaces free from dental biofilm, meaning that over 70% of surfaces are affected by plaque accumulation. Furthermore, it was observed that, in the gingivitis group, the highest plaque accumulation was recorded among 13- and 14-year-old children (χ^2^ = 228,600 *p* < 0.05).

The prevalence of provoked gingival bleeding in the group of children with healthy periodontium had an average value of approximately 6%. In contrast, in the group of children with gingival inflammation, the average value was 69.8%; according to the new classification of periodontal diseases, the latter characterizes generalized gingival inflammation. Significant differences between children with healthy periodontium and those with gingivitis were observed across all age groups (χ^2^ = 157,855 *p* < 0.05).

### 3.3. Total Microbial Load

The following table presents the quantity (total count per sample) of examined subgingival microorganisms in all children included in this study (Table 3).

The average quantity of isolated microorganisms (MO) in children with healthy periodontium was 3.8 × 10^7^ ± 5.9 × 10^7^, while a significantly higher quantity was isolated in children with gingival inflammation: 4.0 × 10^8^ ± 1.1 × 10^9^ (*p* < 0.05).

### 3.4. Relative Proportion of Isolated Microorganisms in Different Groups of Children

**Figure 2:**
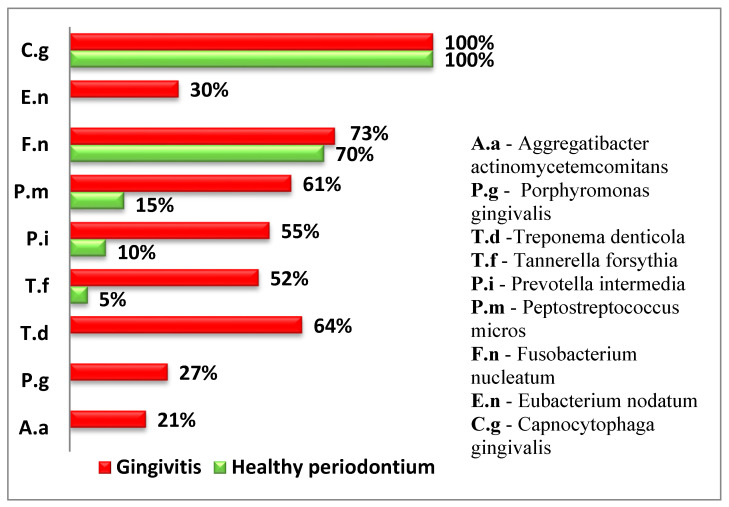
Relative frequency of isolated subgingival microorganisms (% of samples with positive detection).

To provide a comprehensive overview of the subgingival microorganisms in the study groups, both the frequency of isolation and the average quantity of each microorganism were assessed using two distinct formats. Figure 2 illustrates the relative frequency of detection for each microorganism, expressed as the percentage of samples in which it was isolated. In contrast, Table 4 presents the mean quantitative values (e.g., CFU/mL) of the isolated microorganisms in each group. These two data sets offer complementary information: the frequency of isolation indicates how commonly a microorganism is found among the participants, while the average quantity reflects the microbial load in the positive samples.

The following diagram presents the relative proportion of the examined periodontopathogens in children from both groups (Figure 2).

Four of the examined periodontopathogens (*A. actinomycetemcomitans, P. gingivalis, T. denticola,* and *E. nodatum*) were not isolated in children with healthy periodontium.

In both groups, *T. forsythia*, a representative of the red complex according to Socransky, was isolated. However, in the group of children with gingival inflammation, the frequency of isolation was significantly higher (*p* < 0.05).

Among the microorganisms from the orange complex, *F. nucleatum*, which is the second most frequently isolated microorganism, was found in over 70% of the examined children and was relatively evenly distributed between children with gingival inflammation and healthy periodontium.

*P. intermedia* and *P. micros* were found significantly more frequently in children with gingivitis.

The representative of the green complex was *C. gingivalis* according to Socransky and was present in all examined children and equally identified in both healthy cases and those with gingivitis.

### 3.5. Quantitative Characteristics of Isolated Periodontopathogens by Group

The following table presents the quantities of isolated periodontopathogens in both study groups (Table 4).

In children with healthy periodontium, the isolated periodontopathogen *T. forsythia* from the red complex, which exhibits high virulence, is found not only in isolated cases, but in insignificantly low quantities.

Only *F. nucleatum* and *C. gingivalis* were isolated in higher quantities among children with healthy periodontium.

In children with gingival inflammation, the highest quantities of isolated periodontopathogens were *P. intermedia* and *C. gingivalis*.

Regarding *T. forsythia* from the red complex and *P. Intermedia* and *P. micros* from the orange complex, significantly higher values were recorded in children with gingival inflammation (*p* < 0.05).

## 4. Discussion

The oral cavity is home to hundreds of unique microbial species, with specific periodontopathogens being isolated from different ecological niches [26]. In the context of periodontal pathology, the composition of microbial species in the supra- and subgingival biofilm undergoes dramatic changes [27].

The aim of this study was to isolate and compare key microorganisms from the subgingival microbiota in children with healthy periodontium and children with gingival inflammation between the ages of 10 and 14.

As expected, children with gingival inflammation exhibited poor oral hygiene status and, consequently, a significantly higher microbial load compared to children with healthy periodontium. The relationship between oral hygiene and the consumption of sugar candies should be carefully considered when evaluating the factors contributing to oral health outcomes. Our findings suggest that subgingival microbiome profiling can serve as a valuable tool for the early detection and prevention of gingival inflammation in children. Although the 2017 World Workshop classification defines bleeding on probing (BoP) as a key diagnostic criterion, we applied the papillary bleeding index (PBI) to quantify inflammation severity, particularly suitable for pediatric screening. This approach aligns with the concept of site-specific inflammation and allows for a more nuanced assessment of early gingival changes in children. Microbiological assessment could be applied not only in children presenting clinical signs of gingivitis but also in otherwise healthy children with risk factors, enabling targeted preventive measures and improved oral hygiene management. The formation of subgingival plaque occurs through the coaggregation of microorganisms, meaning that the intake of sugary foods plays a significant role in the formation of supragingival plaque, which, in turn, contributes significantly to the development of subgingival biofilm [28].

Regarding the microbiological findings, four species—*A. actinomycetemcomitans*, *P. gingivalis*, *T. denticola*, and *E. nodatum*—were not isolated in children with healthy periodontium. A study by Papaioannou et al. on 93 healthy children aged 3–12 years found that *A. actinomycetemcomitans* was not isolated in any of the examined samples, while *P. gingivalis* and *T. denticola* were isolated in subgingival samples from children aged 9–12 years [29]. The authors suggest that as age increases, the subgingival microbiota becomes more complex, though this does not necessarily lead to the manifestation of gingival inflammation. A systematic review of the literature shows a significantly higher isolation of *A. actinomycetemcomitans, P. gingivalis*, and *T. denticola* in children with healthy periodontal status. The authors found that highly pathogenic representatives of the red complex according to Socransky *P. gingivalis* were isolated in 29% of the children with healthy periodontium. This is likely related to the age of the children (14–18years) and indicates a trend towards a more complex biofilm composition, without necessarily leading to clinically expressed inflammation [17].

Notably, other studies report *P. gingivalis* in younger children using sensitive genetic methods. For instance, in one study it was detected in 40% of children aged 6–36 months [30], while Umeda et al. reported an 8.9% prevalence of *P. gingivalis* and that of 48.2% for *T. denticola* in 8-year-old Japanese children [31]. The presence of these pathogens in healthy individuals may be transient. Ooshima et al. and Lamell et al. both suggest that species such as *P. gingivalis*, *T. denticola*, and *A. actinomycetemcomitans* may temporarily colonize the oral cavity but not establish persistent infection until later stages of adolescence [32,33].

*F. nucleatum*, the second most prevalent species in our study, was detected in over 70% of all children, and evenly distributed between the groups. Its established role in coaggregation and biofilm maturation may explain this prevalence, as previously demonstrated [33,34]. However, the literature remains divided. While Benitez-Páez et al. found higher proportions of *F. nucleatum* in gingivitis and early periodontitis [35], Wendland et al. reported the opposite trend [36].

*Capnocytophaga gingivalis*, from the green complex, was present in all subjects, regardless of clinical status, consistent with previous findings [35,36,37]. Its presence likely reflects its role in early colonization and biofilm organization rather than direct pathogenicity.

Quantitative analysis revealed significantly higher levels of *P. intermedia* and *C. gingivalis* in the gingivitis group. These findings are supported by studies such as that of Albandar et al., who noted elevated *P. intermedia* in adolescents with active periodontal disease [38,39], as well as associations with systemic conditions such as polycystic ovary syndrome (PCOS) [36].

Furthermore, elevated levels of *T. denticola* were observed in children with gingival inflammation. A study by Marotz et al. identified the interaction between *Treponema* and *Corynebacterium* as a novel microbial indicator for early periodontal deterioration, potentially linked to systemic health markers [40]. The authors determined that this early indicator correlates with poor periodontal health and cardiometabolic markers in the early stages of disease pathogenesis, which should not be underestimated, especially in childhood and adolescence.

Importantly, representatives of the orange and green complexes were found in both groups, suggesting their foundational role in biofilm formation. However, red complex species were found exclusively or in significantly higher prevalence among children with gingival inflammation. *T. forsythia* was detected in healthy children only sporadically and in low quantities.

The red complex has been widely studied for its aggressive virulence and capacity to impair host immunity [40]. Papaioannou et al. reported increased detection of red complex species with age, potentially due to rising plaque levels and gingival inflammation—this observation was echoed in our findings [29]. Nakagawa et al. and Morinushi et al. also noted age-associated increases in IgG antibodies to *P. gingivalis*, further supporting the role of immune maturation in modulating microbial composition [41,42].

Our findings focus on children in puberty and early-onset permanent dentition, a critical developmental window marked by dynamic changes in the oral microbiome. These changes likely contribute to differences in subgingival microbial composition and behavior, which may influence susceptibility to gingival inflammation. Importantly, the presence of typical periodontal pathogens not only in children with early gingival inflammation, but also in systemically healthy children, suggests that microbial shifts precede clinical disease. This insight adds valuable understanding to the early pathogenesis of gingivitis and highlights the need for further longitudinal studies in this population, particularly in understudied regional groups such as Bulgarian children.

## 5. Conclusions

This study provides evidence that children with gingival inflammation exhibit a distinct subgingival microbiome composition compared to children with healthy periodontium. The presence and elevated quantities of specific periodontopathogens—particularly members of the red complex such as *T. forsythia*, *P. gingivalis*, and *T. denticola*—were strongly associated with clinical signs of inflammation. In contrast, these pathogens were absent or only sporadically detected in healthy children, who demonstrated better oral hygiene and lower total microbial load. However, the cross-sectional nature of this study limits the ability to establish causal relationships. Further longitudinal and metagenomic studies are necessary to validate these findings and to better understand the dynamic changes in the pediatric subgingival microbiome and their clinical implications.

## 6. Limitations and Future Considerations

This study has several limitations that should be acknowledged. Firstly, the relatively small sample size may limit the generalizability of the findings to wider pediatric populations. Secondly, the cross-sectional design does not allow for causal inferences regarding the relationship between microbial presence and the onset or progression of gingival inflammation. Furthermore, the limited target panel in these studies does not provide a comprehensive assessment of the subgingival flora, and it would be beneficial for future studies to include a broader diversity of microorganisms. Despite the limitations of this study, our results emphasize the importance of microbiological monitoring in pediatric populations for the better understanding and management of gingival inflammation. Future research employing expanded diagnostic panels and longitudinal designs will facilitate the integration of microbiome analysis into clinical practice, enabling personalized prevention and treatment strategies for periodontal diseases in childhood.

## Figures and Tables

**Table 1:** Distribution of children by dentition, gender, and periodontal status.

	Periodontal Status	Boys Healthy Periodontium		Boys Gingivitis		Girls Healthy Periodontium		Girls Gingivitis		Total	
Dentition		N	%	N	%	N	%	N	%	N	%
Mixed dentition		6	29%	3	14%	8	38%	4	19%	21	100
Permanent dentition		12	23%	17	33%	14	27%	9	17%	52	100
Total		18	25%	20	27%	22	30%	13	18%	73	100

**Table 2:** Oral hygiene status and gingival status—PBI (prevalence) of examined children.

	Status	Oral Hygiene Status				Gingival Status			
		Healthy Periodontium		Gingivitis		Healthy Periodontium		Gingivitis	
Age		*n* Child	Plaque Free Surfaces %	*n* Child	Plaque Free Surfaces %	*n* Child	Bleeding Units %	*n* Child	Bleeding Units %
10 years		8	78%	4	37%	8	7%	4	66%
11 years		6	84%	3	31%	6	7%	3	67%
12 years		12	82%	7	34%	12	7%	7	68%
13 years		8	86%	5	26%	8	4%	5	68%
14 years		6	76%	14	27%	6	6%	14	73%
Total		40	81%	33	30%	40	6%	33	70%
		χ^2^ = 228,600 *p* < 0.05				χ^2^ = 157,855 *p* < 0.05			

**Table 3:** Total microbial count in children from both study groups.

Group	*n* Child	Mean ± SD	Mann–Whitney Test
Healthy periodontium	40	3.8 × 10^7^ ± 5.9 × 10^7^	U = 282,000 *p* < 0.05
Gingivitis	33	4.0 × 10^8^ ± 1.1 × 10^9^	

**Table 4:** Mean quantitative values of isolated microorganisms (e.g., CFU/mL) in children with gingival inflammation and healthy periodontium.

Periodontopathogens	Healthy Periodontium		Gingivitis		Mann–Whitney Test *n*
	*n*	Mean ± SD	*n*	Mean ± SD	
*A. actinomycetemcomitans*	0	0	7	2.5 × 10^3^ ± 9.2 × 10^3^	-
*C. gingivalis*	40	6.9 × 10^4^ ± 1.5 × 10^5^	33	1.7 × 10^5^ ± 4.9 × 10^5^	U = 565,000 *p* > 0.05
*P. gingivalis*	0	0	9	2.4 × 10^4^ ± 6.6 × 10^4^	-
*T. denticola*	0	0	21	3.8 × 10^4^ ± 1.1 × 10^5^	-
*T. forsythia*	2	5.5 × 10^1^ ± 2.4 × 10^2^	17	3.6 × 10^3^ ± 6.4 × 10^3^	U = 338,000 *p* < 0.05
*P. intermedia*	4	1.5 × 10^2^ ± 4.7 × 10^2^	18	1.2 × 10^5^ ± 3.0 × 10^5^	U = 344,000 *p* < 0.05
*P. (micromonas)micros*	6	1.3 × 10^2^ ± 5.0 × 10^2^	20	1.9 × 10^3^ ± 2.8 × 10^3^	U = 321,000 *p* < 0.05
*F. nucleatum*	28	1.2 × 10^4^ ± 3.3 × 10^4^	24	1.7 × 10^4^ ± 3.0 × 10^4^	U = 551,000 *p* > 0.05
*E. nodatum*	0	0	10	1.6 × 10^2^ ± 3.7 × 10^2^	-

## Data Availability

The original contributions presented in this study are included in the article. Further inquiries can be directed to the corresponding author.

## Abbreviations

The following abbreviations are used in this manuscript:

PCR	Polymerase chain reaction
PBI	Papillary bleeding index
HI	Hygiene index
AAP	American Academy of Periodontology
EEP	European Federation of Periodontology
BOP	Bleeding on probing
NGS	Next-generation sequencing
